# Supplementary material for: A Critical View of the Application of the APEX Software (Aqueous Photochemistry of Environmentally-Occurring Xenobiotics) to Predict Photoreaction Kinetics in Surface Freshwaters
Source: Molecules. 2019 Dec 18;25(1):9. doi: 10.3390/molecules25010009 (PMC7017383; doi:10.3390/molecules25010009)
Supplement: Supplementary file 1 [file molecules-25-00009-s001.zip › 3.2.4_gcc-4.4.0.zip!/3.2.4_gcc-4.4.0/3.2.4_gcc-4.4.0/doc/octave/html/interpreter/Terminal-Output.html]

Terminal Output - Untitled


Next: Terminal Input,
Up: Basic Input and Output


---

#### 14.1.1 Terminal Output

Since Octave normally prints the value of an expression as soon as it
has been evaluated, the simplest of all I/O functions is a simple
expression. For example, the following expression will display the
value of pi

```
     pi
          -| pi = 3.1416
```

This works well as long as it is acceptable to have the name of the
variable (or ans) printed along with the value. To print the
value of a variable without printing its name, use the function
`disp`.

The `format` command offers some control over the way Octave prints
values with `disp` and through the normal echoing mechanism.

— Built-in Function: **disp** (x)  
> Display the value of x. For example,
>
> ```
>           disp ("The value of pi is:"), disp (pi)
>           
>                -| the value of pi is:
>                -| 3.1416
> ```
>
> Note that the output from `disp` always ends with a newline.
>
> If an output value is requested, `disp` prints nothing and
> returns the formatted output in a string.
>
>
>
> **See also:** fdisp.

— Command: **format**  
— Command: **format** options  
> Reset or specify the format of the output produced by `disp` and
> Octave's normal echoing mechanism. This command only affects the display
> of numbers but not how they are stored or computed. To change the internal
> representation from the default double use one of the conversion functions
> such as `single`, `uint8`, `int64`, etc.
>
> By default, Octave displays 5 significant digits in a human readable form
> (option short paired with loose format for matrices).
> If `format` is invoked without any options, this default format
> is restored.
>
> Valid formats for floating point numbers are listed in the following
> table.
>
> `short`: Fixed point format with 5 significant figures in a field that is a maximum of 10 characters wide. (default). If Octave is unable to format a matrix so that columns line up on the decimal point and all numbers fit within the maximum field width then it switches to an exponential e format. `long`: Fixed point format with 15 significant figures in a field that is a maximum of 20 characters wide. As with the short format, Octave will switch to an exponential e format if it is unable to format a matrix properly using the current format. `short e` `long e`: Exponential format. The number to be represented is split between a mantissa and an exponent (power of 10). The mantissa has 5 significant digits in the short format and 15 digits in the long format. For example, with the short e format, `pi` is displayed as `3.1416e+00`. `short E` `long E`: Identical to short e or long e but displays an uppercase E to indicate the exponent. For example, with the long E format, `pi` is displayed as `3.14159265358979E+00`. `short g` `long g`: Optimally choose between fixed point and exponential format based on the magnitude of the number. For example, with the short g format, `pi .^ [2; 4; 8; 16; 32]` is displayed as ``` ans = 9.8696 97.409 9488.5 9.0032e+07 8.1058e+15 ``` `long G` `short G`: Identical to short g or long g but displays an uppercase E to indicate the exponent. `free` `none`: Print output in free format, without trying to line up columns of matrices on the decimal point. This also causes complex numbers to be formatted as numeric pairs like this (0.60419, 0.60709) instead of like this 0.60419 + 0.60709i.
>
> The following formats affect all numeric output (floating point and
> integer types).
>
> `+` `+` chars `plus` `plus` chars: Print a + symbol for nonzero matrix elements and a space for zero matrix elements. This format can be very useful for examining the structure of a large sparse matrix. The optional argument chars specifies a list of 3 characters to use for printing values greater than zero, less than zero and equal to zero. For example, with the + "+-." format, `[1, 0, -1; -1, 0, 1]` is displayed as ``` ans = +.- -.+ ``` `bank`: Print in a fixed format with two digits to the right of the decimal point. `native-hex`: Print the hexadecimal representation of numbers as they are stored in memory. For example, on a workstation which stores 8 byte real values in IEEE format with the least significant byte first, the value of `pi` when printed in `native-hex` format is `400921fb54442d18`. `hex`: The same as `native-hex`, but always print the most significant byte first. `native-bit`: Print the bit representation of numbers as stored in memory. For example, the value of `pi` is ``` 01000000000010010010000111111011 01010100010001000010110100011000 ``` (shown here in two 32 bit sections for typesetting purposes) when printed in native-bit format on a workstation which stores 8 byte real values in IEEE format with the least significant byte first. `bit`: The same as `native-bit`, but always print the most significant bits first. `rat`: Print a rational approximation, i.e., values are approximated as the ratio of small integers. For example, with the rat format, `pi` is displayed as `355/113`.
>
> The following two options affect the display of all matrices.
>
> `compact`: Remove extra blank space around column number labels producing more compact output with more data per page. `loose`: Insert blank lines above and below column number labels to produce a more readable output with less data per page. (default).

- Paging Screen Output
